# Audit on Discharge Summaries From General Adult Inpatient Units to Primary Care at Black Country Healthcare NHS Foundation Trust

**DOI:** 10.1192/bjo.2024.550

**Published:** 2024-08-01

**Authors:** Pallavi Chandra, Omair Ahmed, Bhavika Vajawat, Olutobi Ojuawo, Prateek Varshney

**Affiliations:** 1 Black Country Healthcare NHS Foundation Trust, Sandwell, United Kingdom; 2 Oxleas NHS Foundation Trust, London, United Kingdom; 3 Oakeswell Health Centre, Wednesbury, United Kingdom; 4 South London and Maudsley, London, United Kingdom

## Abstract

**Aims:**

Discharge summaries act as a key source of condensed information of inpatient stay as well as follow-up plan. Its timely availability to primary care and other multi-disciplinary teams involved in patient care is vital, especially when patients are being managed out of locality by different teams.

The project aimed at assessing if discharge summaries for General Adult inpatients across all four localities of the Trust was made available and in a timely fashion on patient electronic records as well as to primary care using national guidelines as the standard. Using the same guidelines, it also evaluated the quality of the summaries based on the information contained.

**Methods:**

Data was retrospectively collected in October 2023 for general adult inpatient discharges for the month of January 2023 across all four localities of Black Country Healthcare NHS Foundation Trust. Records for 148 out of the 152 discharges were assessed. Data was collected from electronic patient records Rio and evaluated on Microsoft Excel. The evaluation checked whether discharge summaries were available, duration between discharge and its availability on electronic records as well as contents of summary. Professional Record Standards Body and the RCPsych guidelines were used as standards.

**Results:**

28 of the 148 (18.9%) patients did not have a completed discharge summary. Of these, 14 (9.4%) were out of locality patients. The average duration from discharge to summary being made available was 12.7 days. Most of the summaries contained all relevant information as per guidelines.

**Conclusion:**

The findings were presented to the Trust’s QI committee. It was concluded that while majority patients had a summary made available, there is a need for additional strategies to ensure summaries are available soon after discharge to ensure safe post-discharge care.

It was identified that the bed management team should notify parent teams of admissions and discharges promptly.

The medical secretary is to monitor the admissions register and ensure the junior doctors in the team complete discharge summaries in a timely manner.

Business intelligence team to use clinical coding to identify any missing discharge summaries and provide medical teams with a monthly report in case any are missed by the secretaries.

Once above recommendations are implemented, a re-audit would help to analyse the improvements in practice. The results would also help guide the Trust in developing a policy to harmonise processes across the Trust and thereby ensure safe patient care post-discharge.

